# Efficacy and safety of mesenchymal stem cells in patients with acute ischemic stroke: a meta-analysis

**DOI:** 10.1186/s12883-024-03542-1

**Published:** 2024-01-29

**Authors:** Huanjia Huang, Jian Zhang, Jinmei Lin, Shengliang Shi

**Affiliations:** Department of Neurology, The second Affiliated Hospital of Guangxi Medical University, Nanning City, Guangxi Zhuang Autonomous Region Province China

**Keywords:** Mesenchymal stem cells, Transplantation, Ischemic stroke, Randomized controlled studies, Meta-analysis

## Abstract

**Objective:**

This meta-analysis and systematic review were conducted to comprehensively evaluate the efficacy and safety of mesenchymal stem cells in patients with acute ischemic stroke.

**Method:**

We conducted a manual search of electronic databases, including PubMed, Embase, the Cochrane Library, and Web of Science, with a search deadline set for February 1, 2023. Data analysis was performed using Stata version 15.0.

**Result:**

A total of 9 randomized controlled studies were included, involving a total of 316 people, including 159 mesenchymal stem cells and 147 control groups. Results of meta-analysis: Compared to a placebo group, the administration of mesenchymal stem cells resulted in a significant reduction in the National Institutes of Health Stroke Scale (NIHSS) scores among patients diagnosed with acute ischemic stroke [SMD=-0.99,95% CI (-1.93, -0.05)]. Compared to placebo, barthel index [SMD = 0.48,95% CI (-0.55,1.51)], modified rankin score [SMD = 0.45, 95% CI (1.11, 0.21)], adverse events (RR = 0.68, 95% CI (0.40, 1.17)] the difference was not statistically significant.

**Conclusion:**

Based on current studies, mesenchymal stem cell transplantation can ameliorate neurological deficits in patients with ischemic stroke to a certain extent without increasing adverse reactions. However, there was no significant effect on Barthel index and Modified Rankin score.

**Supplementary Information:**

The online version contains supplementary material available at 10.1186/s12883-024-03542-1.

## Introduction

Ischemic stroke (IS) is a prevalent neurological disorder characterized by a substantial incidence, a high disability rate, frequent recurrence, and numerous associated complications. This condition often leaves individuals with varying degrees of residual dysfunction, imposing a substantial medical and societal burden on both families and communities [1, 2]. Based on partial data, in the year 2020, the age-standardized prevalence rate of stroke stood at 1114.8 per 100,000 individuals. Additionally, the incidence rate was recorded at 246.8 per 100,000, while the fatality rate was 114.8 per 100,000. The annual incidence of severe acute ischemic stroke stood at 270 cases per 100,000 individuals, with a corresponding mortality rate of 26% [1–4]. For patients with acute ischemic stroke, the main treatment at home and abroad is to restore cerebral blood flow through timely reperfusion, save the ischemic tissue and reduce the rate of disability [5]. intravenous thrombolysis (IVT) has been proven to be effective and safe in intravenous thrombolysis (IVT) patients with acute cerebral infarction within 4.5 h of the onset, but it lacks an ideal means for functional recovery after tissue injury [3, 4, 6, 7].

In recent years, stem cell transplantation therapy has been recognized as a unique advantage in the field of ischemic stroke treatment [5, 8]. A large number of studies [9–11, 13, 14] have shown that MSCs transplantation has obvious efficacy in IS animal models, which are mainly evaluated according to the improvement in behavioral and histological aspects. In terms of behavior, the ability to remove adhesions is often used to evaluate the autosensory deficit, the spindle test to evaluate the motor function, the limb placement test to evaluate the motor sensory integrity, the balance beam walking test to evaluate the motor coordination function and the nerve function injury score to evaluate the degree of nerve function deficit. Relevant experiments showed that the nerve function improved significantly after MSCs transplantation [12, 13, 15]. In terms of histology, the infarct volume was observed by magnetic resonance imaging or TTC staining. In numerous studies, the infarct volume was consistently found to be significantly diminished in the group treated with Mesenchymal Stem Cells (MSCs) in comparison to the model group [14, 16]. However, there are still many controversies regarding the treatment of acute ischemic stroke by mesenchymal stem cells in clinical practice [15, 17]. Therefore, this meta-analysis is expected to solve the above-mentioned controversies and provide a new treatment option for clinicians and patients.

## Methods

The protocol has been duly registered in the International Prospective Register of Systematic Reviews (PROSPERO) database under the registration number CRD42023407508.

### Retrieval strategy

Search PubMed, Embase, Cochrane library, Web of science for randomized controlled articles on mesenchymal stem cell therapy for acute ischemic stroke published as of February 1, 2023. The search term was (mesenchymal stem cells, acute ischemic stroke). For specific search strategies (Table S1).

### Inclusion and exclusion criteria

The included population met diagnostic criteria for acute ischemic stroke [16, 18]. The experimental group received mesenchymal stem cell intervention and the control group received placebo treatment. The main outcome indexes were BI: Barthel index; NIHSS: National Institute of Health Stroke Scale; mRS: Modified Rankin score; Secondary outcome indicators were adverse reactions. The type of study included was randomized controlled. Exclusion criteria are: conference abstracts, literature reviews, meta-analyses, duplicate publications, animal experiments, case reports, conference abstracts, unavailable full text and unavailable data will be excluded.

### Data extraction

Two separate reviewers conducted an independent assessment of the literature to facilitate data extraction. By reviewing the title, abstract, and full text of the literature, we conducted a direct screening of papers that were readily assessable for inclusion. To incorporate literature into the review, it is essential to refer to the opinions of relevant experts and assess the full-text articles by direct downloading and careful examination. Throughout the screening phase, it is imperative to adhere rigorously to the predefined inclusion and exclusion criteria. Extract relevant observational metrics from both sets of studies and perform a meticulous cross-verification of the gathered data to guarantee data consistency. In the process of data extraction, the primary components encompassed the following: the name of the initial author, publication year, country of origin, type of mesenchymal stem cells, dosage of transplanted stem cells, sample size, gender distribution, age demographics, intervention methodologies, and outcome metrics.

### Risk of bias evaluate

The quality of the studies included in this review was independently assessed by two researchers. We utilized the bias analysis tool outlined in the Cochrane Handbook for Systematic Reviews of Interventions 5.1.0 to evaluate the quality of these included studies [6, 17, 19]. The evaluation included seven aspects: random sequence generation (selectivity bias), assignment concealment (selectivity bias), implementor and participant blinding (implementation bias), outcome evaluator blinding (observation bias), data results integrity (follow-up bias), selective reporting of study results (reporting bias), and other sources of bias. The seven projects were individually assessed in accordance with the aforementioned criteria, with the aim of conducting a comprehensive quality evaluation of the incorporated studies. This process involved generating a methodological quality assessment table, a bias risk graph, and a summary chart of bias risk.

### Data analysis

The collected data were entered into Stata 15.0 software (StataCorp, College Station, TX, USA) for the purpose of conducting statistical analyses. The assessment of heterogeneity was performed using either the I^2^ statistic or the Q statistic. I^2^ values of 0%, 25%, 50%, and 75% correspond to the absence of heterogeneity, minimal heterogeneity, moderate heterogeneity, and substantial heterogeneity, respectively. When the I^2^ statistic was equal to or greater than 50%, we conducted a sensitivity analysis to investigate potential origins of heterogeneity. When heterogeneity was less than 50%, we employed a fixed-effects model. Furthermore, we utilized both the random-effects model and conducted the Egger test to assess the presence of publication bias.

## Results

### Literature screening

**Fig. 1:**
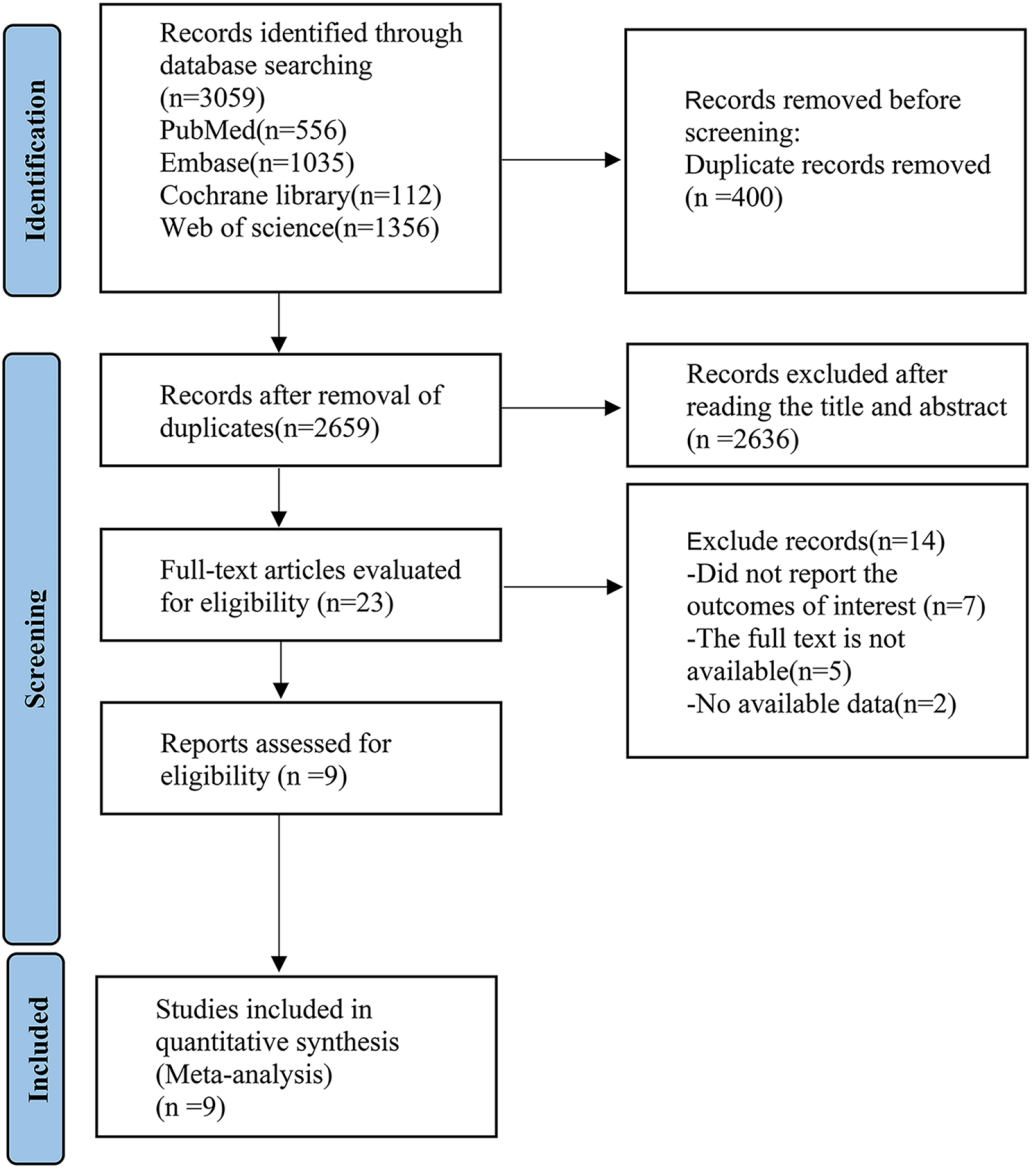
PRISMA flow diagram of the study process. PRISMA, Preferred Reporting Items for Systematic review and Meta-analysis

Using manual retrieval, we obtained a grand total of 3,059 articles, which reduced to 2,659 articles once duplicates were removed. Further scrutiny of titles and abstracts narrowed the selection down to 23 articles. Ultimately, following a thorough review of the full-text versions, 9 articles [18–28] were included in the analysis (see Fig. 1).

### The basic characteristics table of included literature

A total of nine randomized controlled studies were incorporated in this analysis, encompassing a combined study population of 316 participants. Of these, 159 received mesenchymal stem cell (MSC) therapy, while 147 constituted the control groups. Specifically, eight of the studies [20, 21, 23–28, 18, 19, 21–26] utilized bone marrow-derived mesenchymal stem cells, while one study [20, 22] employed adipose-derived mesenchymal stem cells. The administered doses of mesenchymal stem cells across these studies varied, ranging from 1 × 10^5^ to 5 × 10^7^ cells per treatment. Further details regarding the specific characteristics of these studies can be found in Table S2.

### Risk of bias assessment

**Fig. 2:**
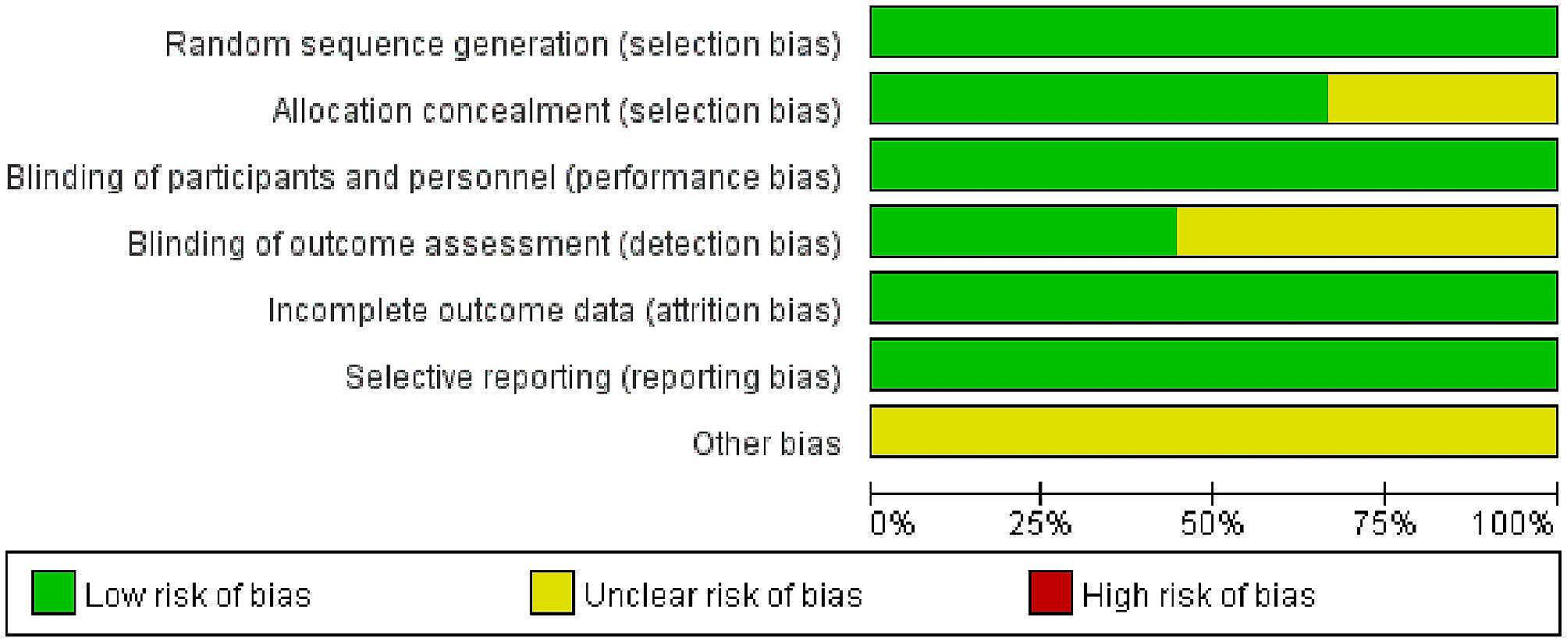
Risk bias of graph

**Fig. 3:**
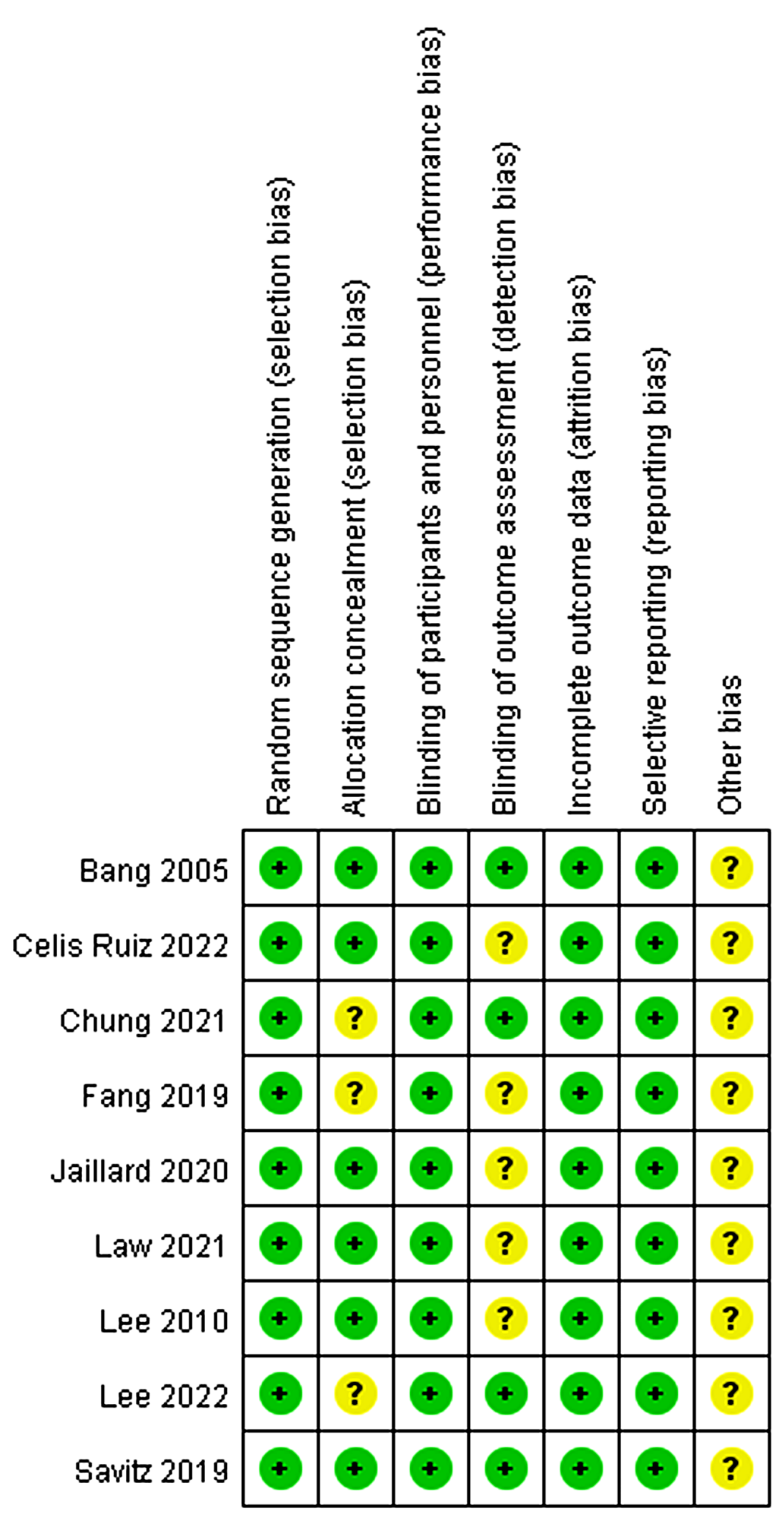
Risk bias of summary

In this study, we examined nine articles, all of which provided comprehensive explanations regarding their randomization methods and the use of blinding. Among these articles, four [18–21, 25–28] also detailed the blinding techniques employed for outcome evaluators. The assessment of bias risk in these articles is visually depicted in Figs. 2 and 3.

### Meta analysis of Barthel index

A total of 3 articles [18, 20, 22–25] mentioned the Barthel index, involving a total of 78 people, including 38 people in the mesenchymal stem cell group and 40 people in the placebo group, and heterogeneity test (I^2^ = 73.2%, *P* = 0.024). Therefore, random effects model was adopted to analyze the included studies. Analysis results [SMD = 0.48,95%CI (-0.55,1.51)] suggested that compared with placebo, mesenchymal stem cells had no significant statistical significance for Barthel index in patients with acute ischemic stroke (Figure S1). In cases where heterogeneity exceeded 50%, a sensitivity analysis was undertaken. The outcomes of this analysis demonstrated that the index’s sensitivity was minimal, ensuring the stability of the results (Figure S2).

### Meta analysis of National Institute of Health Stroke Scale

A total of 4 articles [7–10, 20, 22, 24, 25, 7–9, 27, 18, 20, 22, 23] mentioned the National Institute of Health Stroke Scale, involving a total of 97people, including 39 people in the mesenchymal stem cell group and 58 people in the placebo group, and heterogeneity test (I^2^ = 73.2%, *P* = 0.011). Therefore, random effects model was adopted to analyze the included studies. Analysis results [SMD=-0.99,95%CI (-1.93, -0.05)] suggested suggest that compared with placebo, mesenchymal stem cells can significantly reduce the National Institute of Health Stroke Scale in patients with acute ischemic stroke (Figure S3). In cases where heterogeneity exceeded 50%, a sensitivity analysis was performed. The findings from this analysis revealed that the index’s sensitivity was low, and the results of the analysis demonstrated stability (refer to Figure S4).

### Meta analysis of modified Rankin score

A total of 4 articles [18, 20, 22–25, 27] mentioned modified Rankin score, involving a total of 130 people, including 46 people in the mesenchymal stem cell group and 84 people in the placebo group, and heterogeneity test (I^2^ = 62.5%, *P* = 0.046). Therefore, random effects model was adopted to analyze the included studies. Analysis results [SMD=-0.45,95%CI (-1.11, 0.21)] suggested that compared with placebo, mesenchymal stem cells had no significant statistical significance for modified Ranking score in patients with acute ischemic stroke (Figure S5).In response to heterogeneity exceeding 50%, a sensitivity analysis was performed, revealing that the sensitivity of this parameter was minimal, and the analysis findings remained consistent (Figure S6).

### Meta analysis of adverse events

A total of 5 articles [19–26, 28] mentioned adverse events, involving a total of 166 people, including102 people in the mesenchymal stem cell group and 64 people in the placebo group, and heterogeneity test (I^2^ = 56.4%, *P* = 0.057). Therefore, random effects model was adopted to analyze the included studies. Analysis results [RR = 0.68,95%CI (0.40, 1.17)] suggested that compared with placebo, mesenchymal stem cells had no significant statistical significance for adverse events in patients with acute ischemic stroke (Figure S7).

### Publication bias

The Egger test was employed to assess the publication bias of the included metrics, including the National Institute of Health Stroke Scale, Modified Rankin Score, and adverse events. Results (National Institute of Health Stroke Scale *P* = 0.352, Modified Rankin score *P* = 0.829, adverse events *P* = 0.280) There is no publication bias in these indicators (Figure S8-S10).

## Discussion

Mesenchymal stem cells were first described by FRIEDENSTEIN in 1970 as spindle bone marrow stromal cells attached to plastics [28, 29]. In 1991, CAPLAN [29, 30] coined the term “mesenchymal stem cells” and predicted that these mesodermal derived cells would become the preferred method of autologous therapy for regenerative purposes. Following developments in recent decades, mesenchymal stem cells have been widely used in clinical trials and to treat a variety of diseases, including blood disorders, graft-versus host diseases, autoimmune diseases, and so on. Even severe cases of novel coronavirus pneumonia [30, 31].

In this study, mesenchymal stem cell transplantation can improve the neurological deficits in ischemic stroke patients to a certain extent. Studies have shown that the therapeutic mechanism of MSCs mainly involves several aspects: (1) immune regulation: Transplanted MSCs mediate immunosuppression, regulate the activation of inflammatory cytokines and microglia through CD200, an anti-inflammatory cytokine overexpressed by stem cells in hypoxic environments, and reduce the number of microglia/macrophages [30–32]. Or induce microglia to become M2-type polarized, which inhibits inflammatory response, so as to reduce nerve damage in stroke rats [32, 33]. (2) Apoptosis: calcineurin IS a kind of threonine/phosphatase, which plays an important role in neurohomeostasis. Is induces the overradicalization of calcineurin to trigger apoptosis signals. MSCs transplantation can inhibit apoptosis by reducing the expression of calcineurin in neurons. Stem cell transplantation may also reduce secondary cell death by inhibiting inflammation [33–35]. (3) Neuron damage: Neuron damage can be caused by a variety of mechanisms, calcium overload, oxidative stress and other mechanisms play an important role in IS induced neuron damage. The transplanted MSCs can relieve calcium overload by upregulating the expression of calcium pump SPCA1, which is mainly located in the Golgi apparatus, reduce mitochondrial dysfunction and enhance antioxidant effect by upregulating an antioxidant enzyme UBIAD1. In addition, MSCs can affect the protein clearance pathway after injury. Inhibit the conversion of ubiquitin-proteasome pathway to autophagy pathway and play a role in limiting neuronal damage p [35–37]. (4) Neuronutrition: MSCs can induce the expression of brain-derived neurotrophic factor (BDNF), vascular endothelial growth factor (VEGF), hepatocyte growth factor and other cytokines, nourish nerves, improve the survival rate of neurons in infarction area, and promote the neuroprotective effect [37, 38]. (5) Endogenous neurogenesis: By increasing the expression of chemokines and polysialase, the transplanted MSCs can increase the migration of endogenous neural progenitors, promote the proliferation of endogenous oligodendrocyte progenitors, promote myelin formation, trigger the formation of nerve cells and enhance the function of neurons. It can down-regulate the inhibitory factor Nogo-A which inhibits axon growth and neuron regeneration and promote neurogenesis [12, 38–40].

Although similar study [40] have been conducted before, more Chinese articles were included in this study, and blinding method was rarely mentioned in the included articles, and the time interval was longer. Moreover, the theory obtained in this study is inconsistent with the previous research, which was disturbed by the Chinese research, so our results are more credible.

Although this study found that mesenchymal stem cells can improve NIHSS (National Institute of Health Stroke Scale) in patients with acute ischemic stroke, it still has the following limitations: first, the number of included studies is small and the number of people included in each study is small, which may affect the study; second, the dosage of mesenchymal stem cells used in included studies is inconsistent. Thirdly, transplantation methods and time window of mesenchymal stem cells were included in the study, and due to the small sample size, subgroup analysis could not be further conducted according to different transplantation methods, time window and number of transplanted cells.

## Conclusion

Based on current studies, mesenchymal stem cell transplantation can ameliorate neurological deficits in patients with ischemic stroke to a certain extent without increasing adverse reactions. However, there was no significant effect on Barthel index and Modified Rankin score. However, due to the limitations of the study, more high-quality and large sample studies are expected to prove our conclusion in future studies.

### Electronic supplementary material

Below is the link to the electronic supplementary material.

Supplementary Material 1

## Data Availability

All the data involved in the literature are available.

## Abbreviations

IS: Ischemic stroke
IVT: Intravenous thrombolysis
MSCs: Mesenchymal stem cells
BI: Barthel index
NIHSS: National Institute of Health Stroke Scale
mRS: Modified Rankin score
