# Supplementary material for: Efficacy and safety of mesenchymal stem cells in patients with acute ischemic stroke: a meta-analysis
Source: BMC Neurol. 2024 Jan 29;24:48. doi: 10.1186/s12883-024-03542-1 (PMC10823675; doi:10.1186/s12883-024-03542-1)
Supplement: Supplementary file 1 — Supplementary Material 1 [file 12883_2024_3542_MOESM1_ESM.docx]

**Table S1** The searching strategy

| Search number | Query |
| --- | --- |
| 1 | " Mesenchymal Stem Cells "[Mesh] |
| 2 | ((((((((((((Stem Cell, Mesenchymal[Title/Abstract]) OR (Mesenchymal Stem Cell[Title/Abstract])) OR (Stem Cells, Mesenchymal[Title/Abstract])) OR (Bone Marrow Mesenchymal Stem Cells[Title/Abstract])) OR (Bone Marrow Stromal Cells[Title/Abstract])) OR (Bone Marrow Stromal Cells, Multipotent[Title/Abstract])) OR (Adipose Derived Mesenchymal Stem Cells[Title/Abstract])) OR (Mesenchymal Stem Cells, Adipose Derived[Title/Abstract])) OR (Adipose Tissue-Derived Mesenchymal Stem Cell[Title/Abstract])) OR (Mesenchymal Stromal Cells[Title/Abstract])) OR (Multipotent Mesenchymal Stromal Cells[Title/Abstract])) OR (Mesenchymal Progenitor Cells [Title/Abstract])) OR (Wharton's Jelly Cell[Title/Abstract]) |
| 3 | 1 OR 2 |
| 4 | "Ischemic Stroke"[Mesh] |
| 5 | "Cerebral Infarction"[Mesh] |
| 6 | ((((((((((((((((Ischemic Stroke[Title/Abstract]) OR (Ischemic Strokes[Title/Abstract])) OR (Stroke, Ischemic[Title/Abstract])) OR (Ischaemic Stroke[Title/Abstract])) OR (Ischaemic Strokes[Title/Abstract])) OR (Stroke, Ischaemic[Title/Abstract])) OR (Cryptogenic Ischemic Stroke[Title/Abstract])) OR (Cryptogenic Ischemic Strokes[Title/Abstract])) OR (Ischemic Stroke, Cryptogenic[Title/Abstract])) OR (Cerebral infarction[Title/Abstract])) OR (Cerebral Infarctions[Title/Abstract])) OR (Infarctions, Cerebral[Title/Abstract])) OR (Infarction, Cerebral[Title/Abstract])) OR (Cerebral Infarct[Title/Abstract])) OR (Cerebral Infarcts[Title/Abstract])) OR (Infarct, Cerebral[Title/Abstract])) OR (Infarcts, Cerebral[Title/Abstract]) |
| 7 | 4 OR 5 OR 6 |
| 8 | 3 AND 7 |

**Table S2** The specific features of the paper

| Study | Year | Country | Stem cell species | Stem cell dose | Sample size | | Gender(M/F) | Mean age (years) | | Intervention | | Outcome |
| --- | --- | --- | --- | --- | --- | --- | --- | --- | --- | --- | --- | --- |
|  |  |  |  |  | EG | CG |  | EG | CG | EG | CG |  |
| Bang | 2005 | Korea | BMSCs | 1X106 | 5 | 25 | 18/12 | 63 | 59.3 | BMSCs | Placebo | F1; F2; F3 |
| Lee | 2010 | Korea | BMSCs | 5X107 | 16 | 36 | 34/18 | 64 | 64.9 | BMSCs | Placebo | F3 |
| Fang | 2019 | China | BMSCs | 1X106 | 5 | 6 | 9/2 | 49.4 | 52.83 | BMSCs | Placebo | F1; F2; F3; F4 |
| Savitz | 2019 | USA | BMSCs | 1X107 | 29 | 19 | 35/13 | 59.3 | 62.9 | BMSCs | Placebo | F4 |
| Jaillard | 2020 | France | BMSCs | 1X106 | 16 | 15 | 22/9 | 55 | 53 | BMSCs | Placebo | F1; F2; F3; F4; F5 |
| Law | 2021 | Malaysia | BMSCs | 2x106 | 9 | 8 | 10/7 | 54.6 | 64 | BMSCs | Placebo | F1; F2; F3; F4 |
| Celis Ruiz | 2022 | Spain | ADMSCs | 1X107 | 9 | 10 | 4/15 | 78 | 76 | ADMSCs | Placebo | F2; F3; F4 |
| Lee | 2022 | Korea | BMSCs | 1X107 | 31 | 13 | 24/20 | 63.4 | 61.5 | BMSCs | Placebo | F5 |
| Chung | 2021 | Korea | BMSCs | 1X107 | 39 | 15 | 27/27 | 63.03 | 64.27 | BMSCs | Placebo | F4 |

BMSCs: Bone marrow mesenchymal stem cells; ADMSCs: Adipose Tissue Derived Mesenchymal Stem Cells; F1: BI:Barthel index; F2: NIHSS: National Institute of Health Stroke Scale; F3: mRS: Modified Rankin score; F4: adverse events; F5: FMA: Fugl-Meyer assessmen.


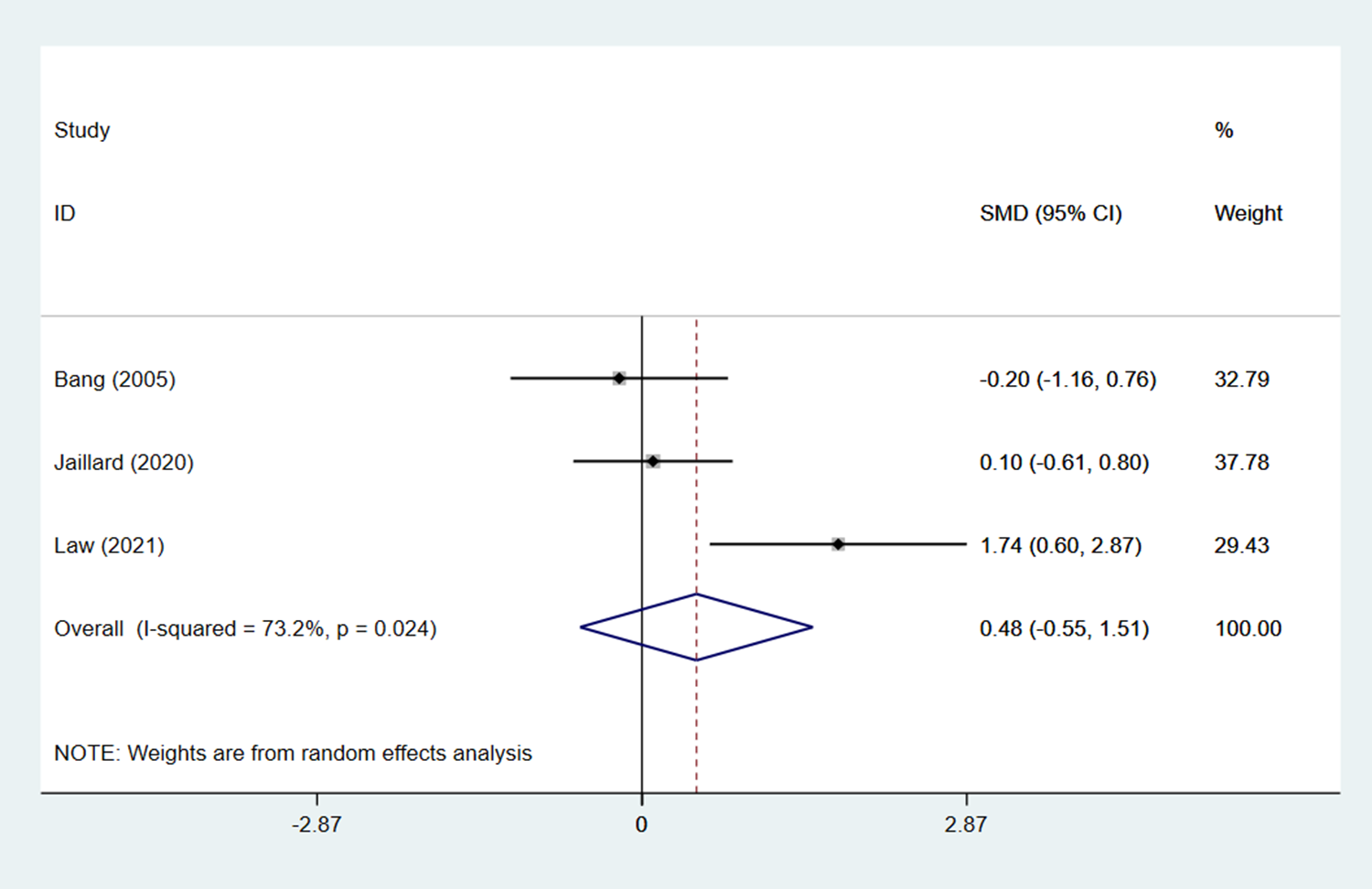


**Figure S1** Meta analysis of Barthel index


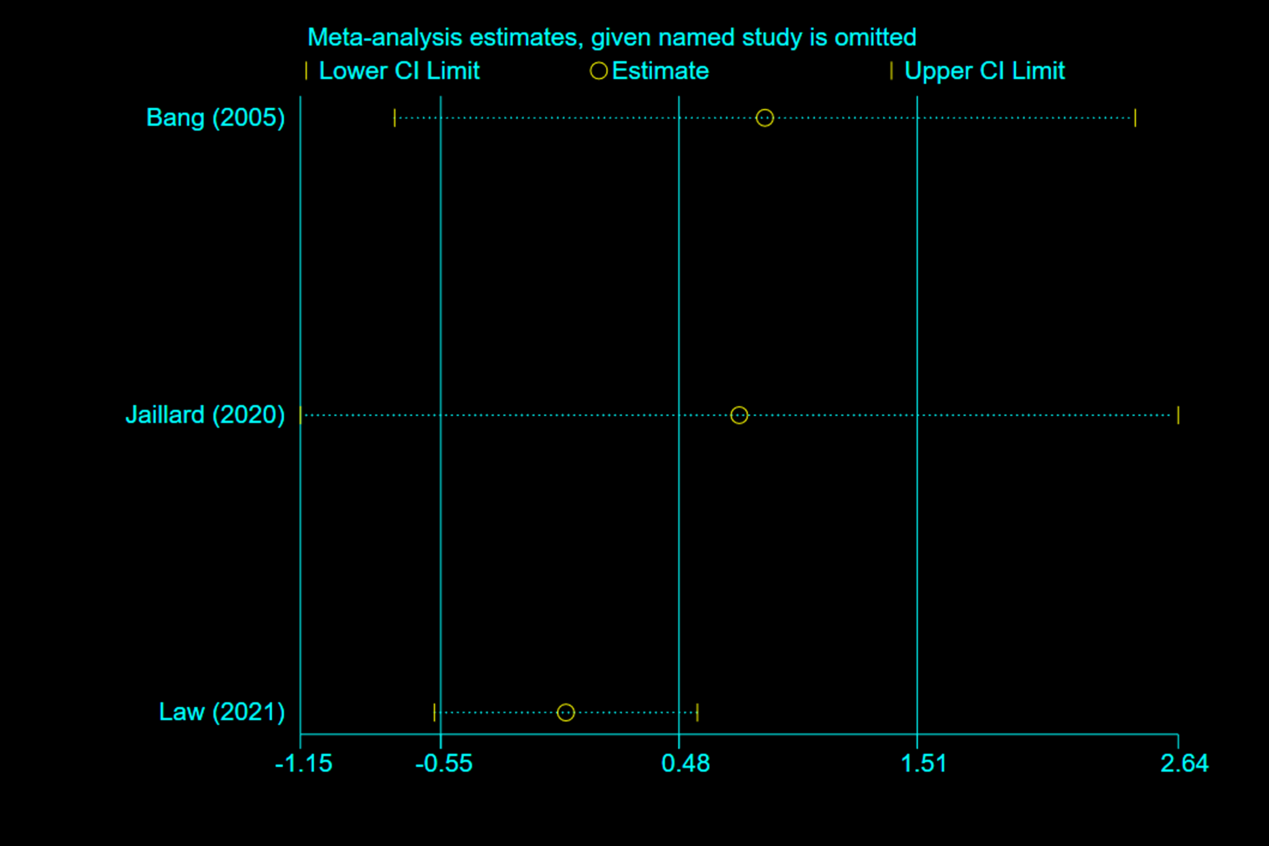


**Figure S2** Barthel index sensitivity analysis


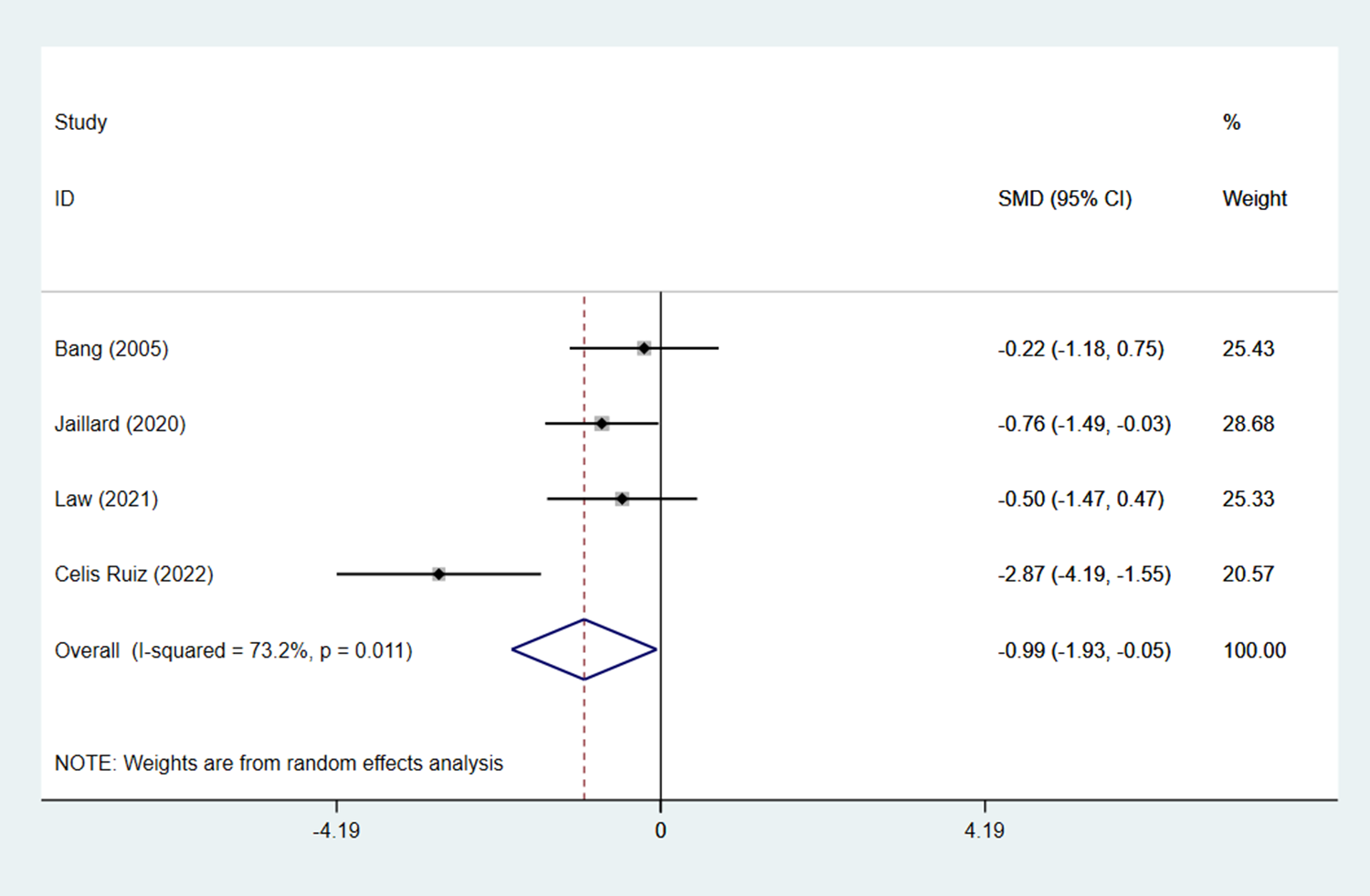


**Figure S3** Meta analysis of National Institute of Health Stroke Scale


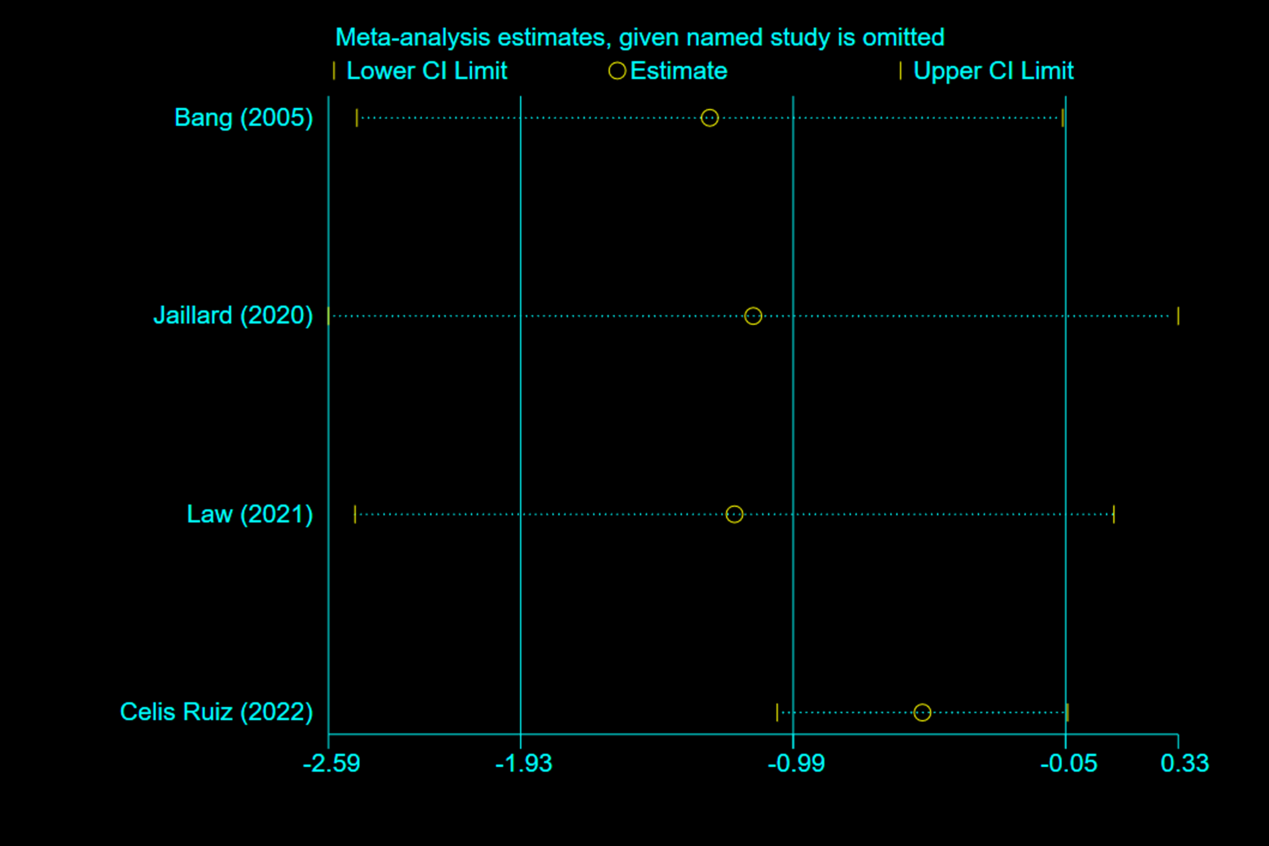


**Figure S4** National Institute of Health Stroke Scale sensitivity analysis


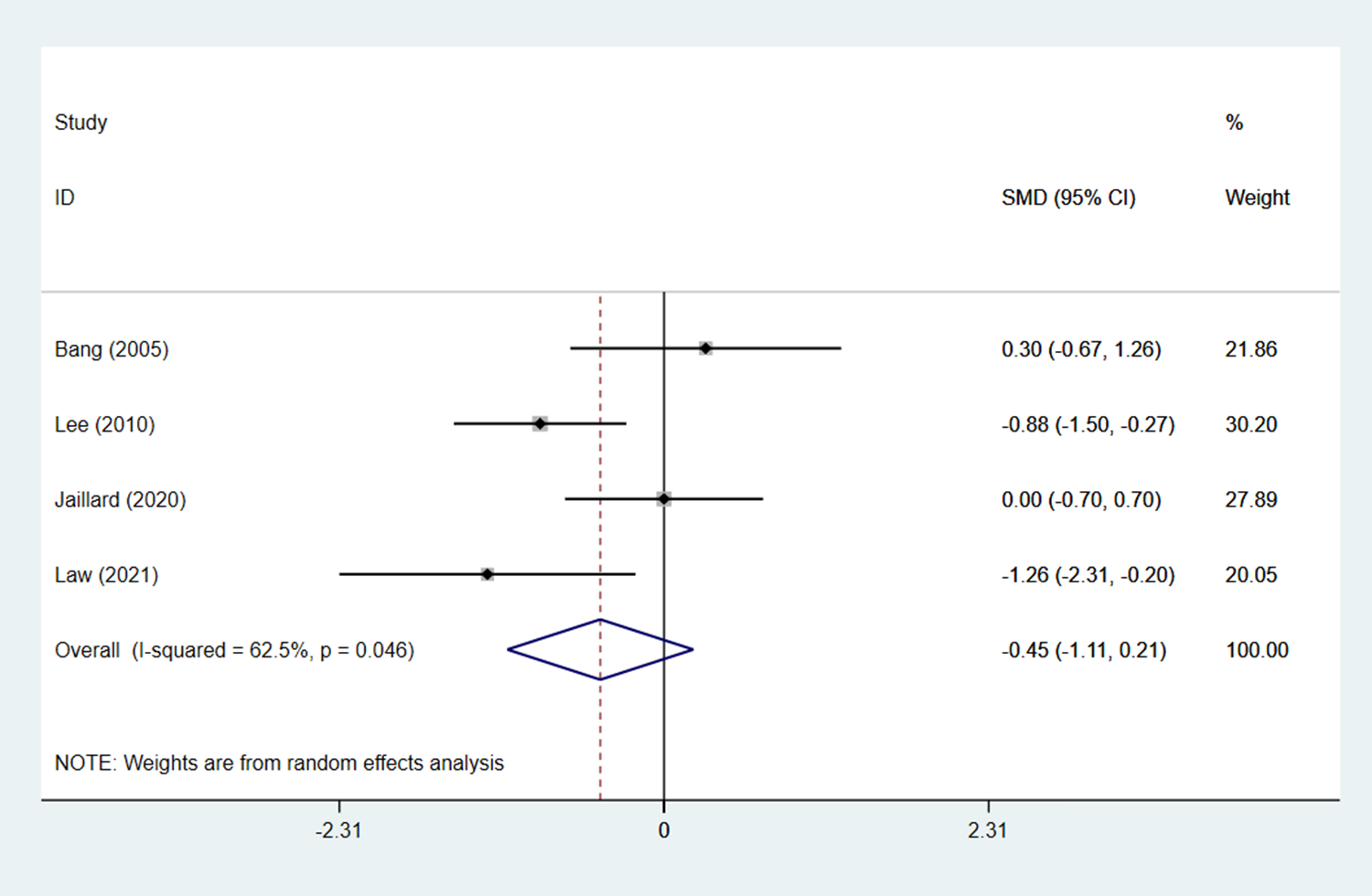


**Figure S5** Meta analysis of Modified Rankin score
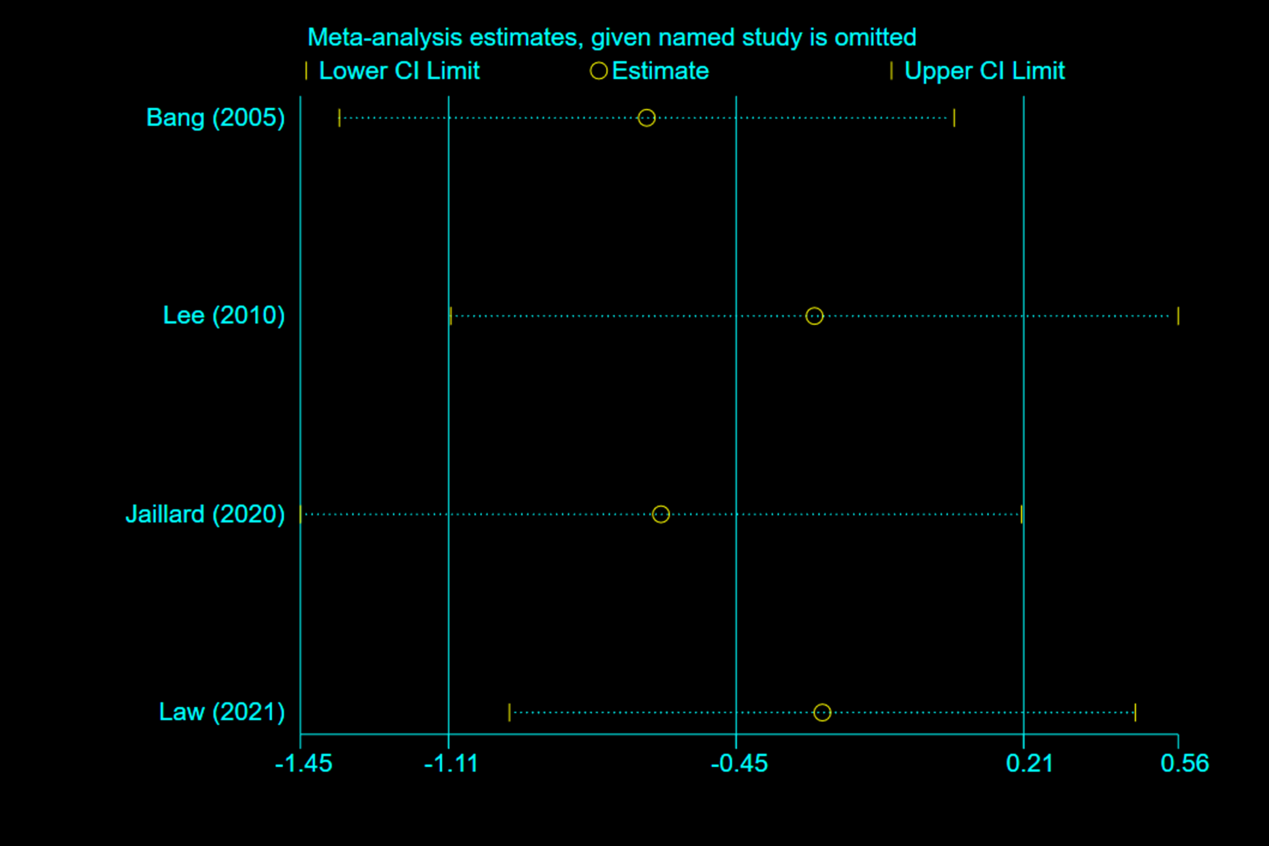


**Figure S6** Modified Rankin score sensitivity analysis


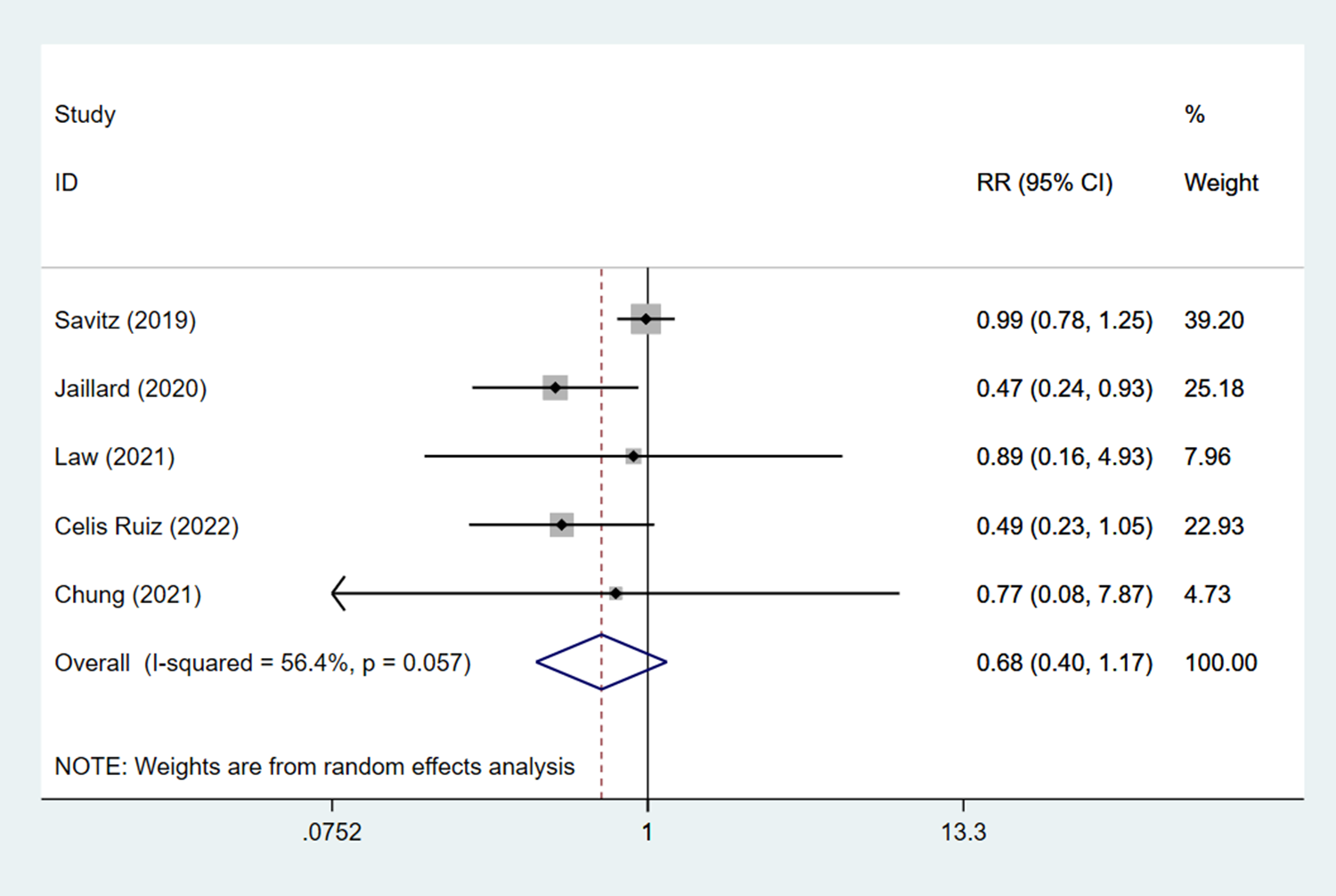


**Figure S7** Meta analysis of adverse events


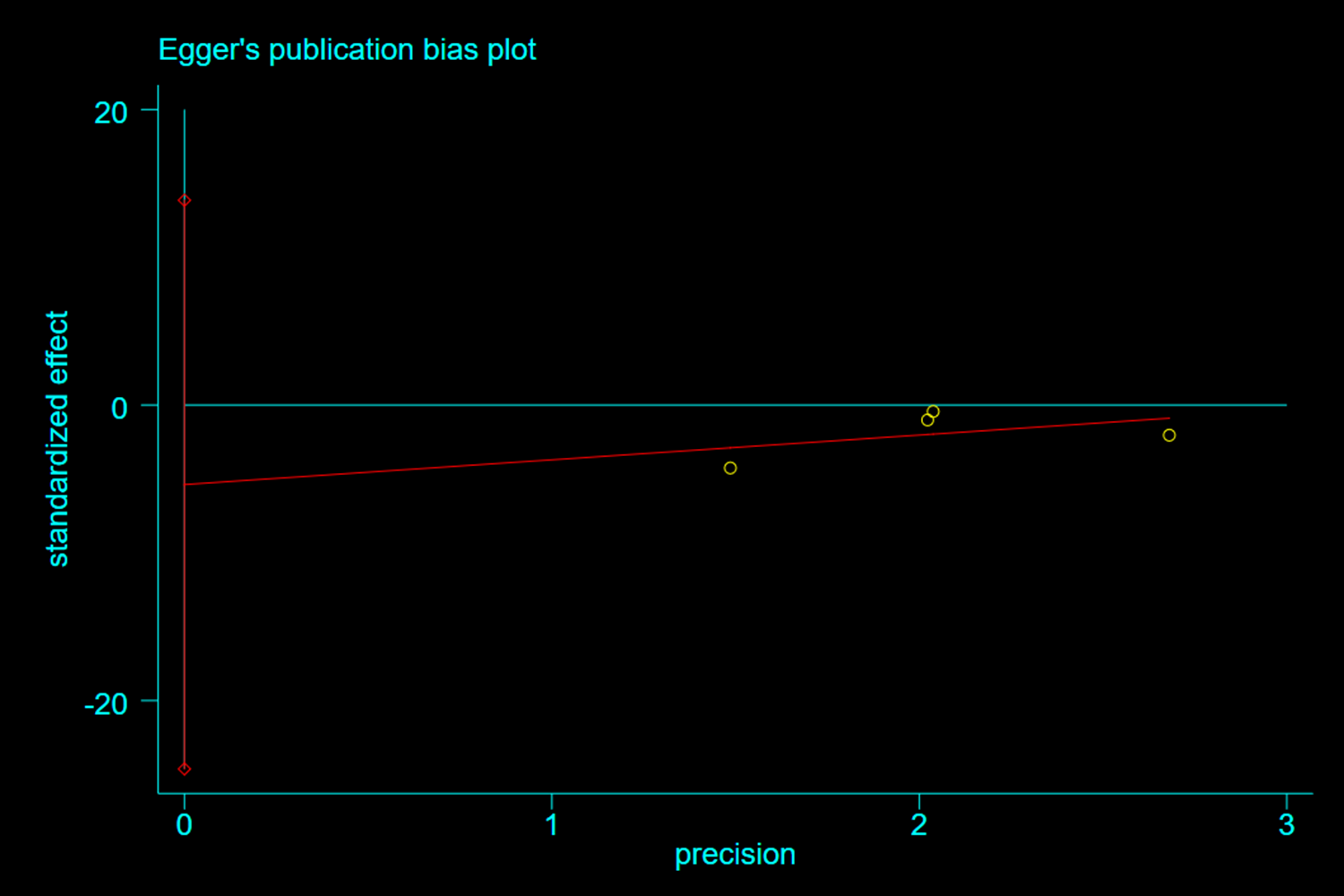


**Figure S8** National Institute of Health Stroke Scale egger test

**
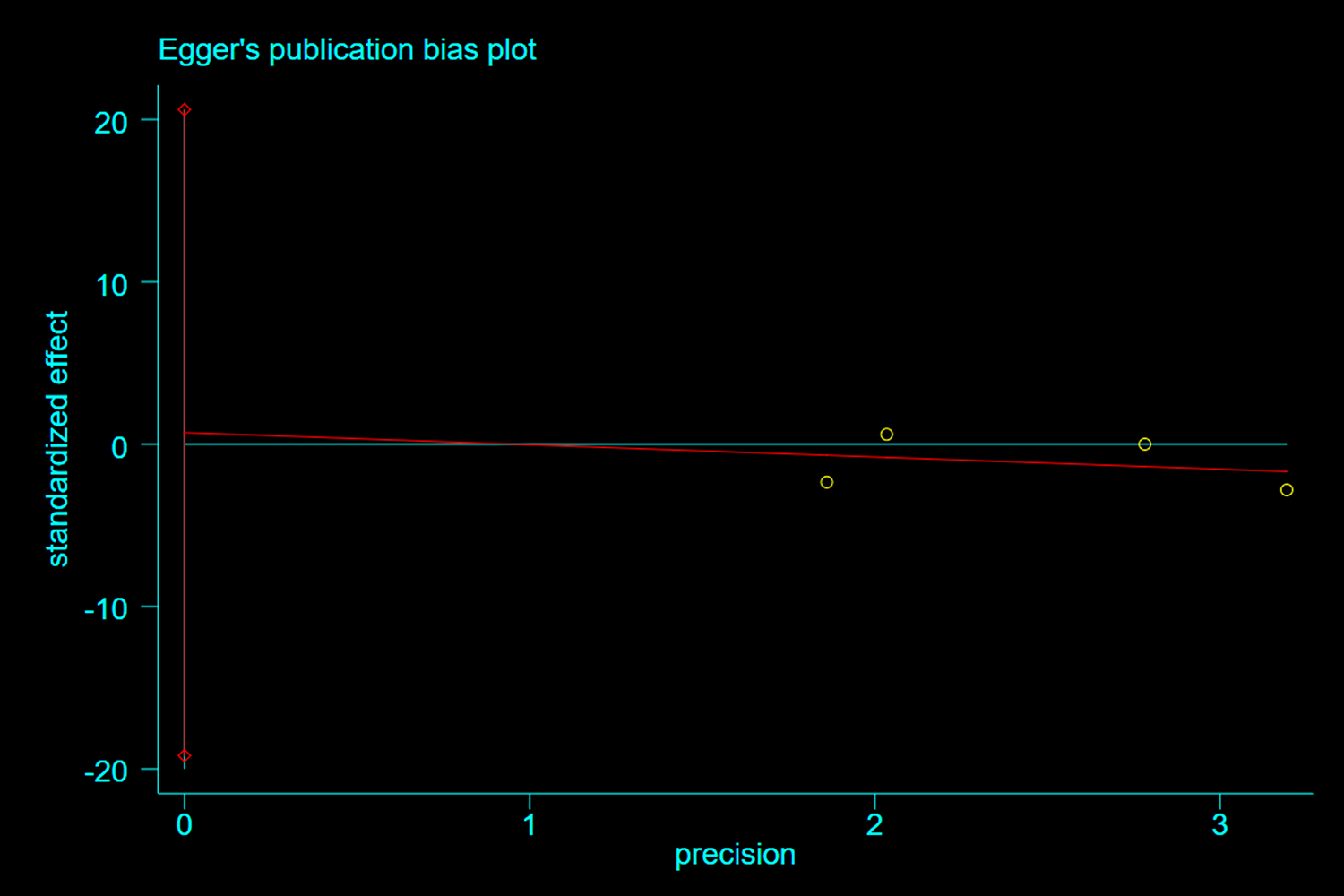
**

**Figure S9** Modified Rankin score egger test


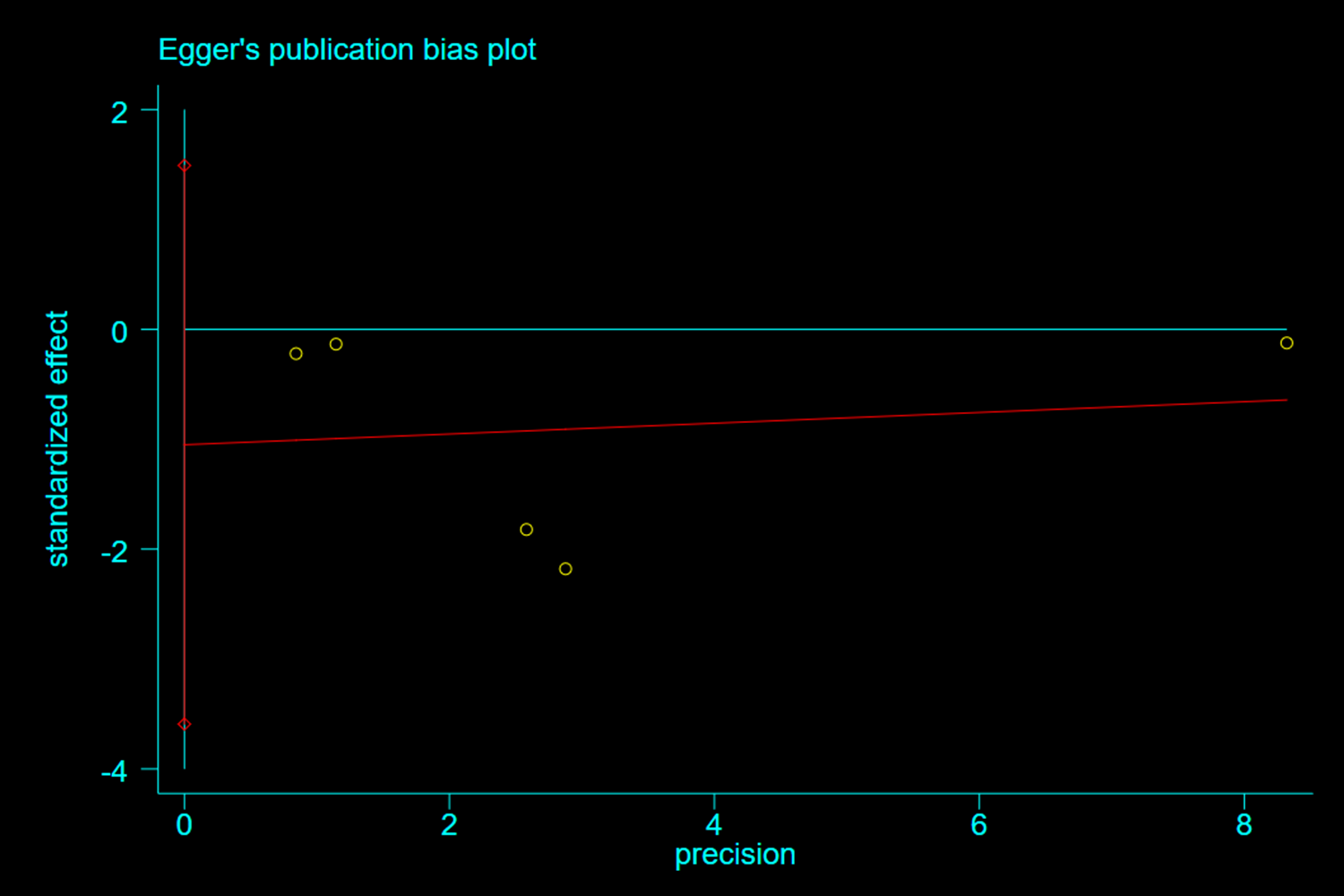


**Figure S10** Adverse events egger test
